# Higher abundance of enterovirus A species in the gut of children with islet autoimmunity

**DOI:** 10.1038/s41598-018-38368-8

**Published:** 2019-02-11

**Authors:** Ki Wook Kim, Jessica L. Horton, Chi Nam Ignatius Pang, Komal Jain, Preston Leung, Sonia R. Isaacs, Rowena A. Bull, Fabio Luciani, Marc R. Wilkins, Jacki Catteau, W. Ian Lipkin, William D. Rawlinson, Thomas Briese, Maria E. Craig

**Affiliations:** 1 School of Women’s and Children’s Health, University of New South Wales Faculty of Medicine, Sydney, Australia; 2 Virology Research Laboratory, Prince of Wales Hospital Randwick, Sydney, Australia; 3 School of Biotechnology and Biomedical Sciences, University of New South Wales Faculty of Science, Sydney, Australia; 4 Center for Infection and Immunity, Mailman School of Public Health, Columbia University, New York, USA; 5 Systems Medicine, Inflammation and Infection Research Centre, School of Medical Sciences, University of New South Wales Faculty of Medicine, Sydney, Australia; 6 Institute of Endocrinology and Diabetes, Children’s Hospital at Westmead, Sydney, Australia; 7 Department of Pathology and Neurology, College of Physicians & Surgeons, Columbia University, New York, USA; 8 Serology and Virology Division, South Eastern Area Laboratory Services Microbiology, Prince of Wales Hospital, Sydney, Australia; 9 Department of Epidemiology, Mailman School of Public Health, Columbia University, New York, USA; 10 Discipline of Child and Adolescent Health, University of Sydney, Sydney, Australia

## Abstract

Enteroviruses (EVs) are prime candidate environmental triggers of islet autoimmunity (IA), with potential as vaccine targets for type 1 diabetes prevention. However, the use of targeted virus detection methods and the selective focus on EVs by most studies increases the risk for substantial investigation bias and an overestimated association between EV and type 1 diabetes. Here we performed comprehensive virome-capture sequencing to examine all known vertebrate-infecting viruses without bias in 182 specimens (faeces and plasma) collected before or at seroconversion from 45 case children with IA and 48 matched controls. From >2.6 billion reads, 28 genera of viruses were detected and 62% of children (58/93) were positive for ≥1 vertebrate-infecting virus. We identified 129 viruses as differentially abundant between the gut of cases and controls, including 5 EV-A types significantly more abundant in the cases. Our findings further support EV’s hypothesised contribution to IA and corroborate the proposal that viral load may be an important parameter in disease pathogenesis. Furthermore, our data indicate a previously unrecognised association of IA with higher EV-A abundance in the gut of children and provide a catalog of viruses to be interrogated further to determine a causal link between virus infection and type 1 diabetes.

## Introduction

Among the spectrum of environmental factors associated with type 1 diabetes, viruses are postulated to contribute most significantly. Indeed, enterovirus (EV) infection often precedes islet autoimmunity (IA), and rates of EV infection are significantly higher in individuals at type 1 diabetes onset compared to controls^1–4^. Furthermore, systematic review and meta-analysis of 26 studies with over 4,400 participants confirm a significant epidemiological association between EV and type 1 diabetes^5^.

Mostly transmitted faeco-orally, classical EVs establish primary infection in the gut, providing a potential reservoir for spread into the pancreas^6,7^. There, EVs can directly infect and damage pancreatic β-cells^8^. This is consistent with the recent detection of EV protein and/or RNA in the pancreata of 6 adults, biopsied within 9 weeks following type 1 diabetes diagnosis^9^. Additionally, persistent EV infection is associated with increased intestinal permeability and inflammation; both pronounced in mice and children with type 1 diabetes^10^. Alternatively, there is potential for molecular mimicry between EV peptides and islet antigens^11^, which could induce a cross-reactive autoimmune response^12^. However, the use of targeted virus detection methods and the selective focus on EVs by most studies increases the risk for substantial investigation bias and an overestimated association with type 1 diabetes. Therefore, an unbiased examination of all viruses (the ‘virome’) is needed to re-evaluate existing associations.

Previously, the Finnish Diabetes Prediction and Prevention (DIPP) and The Environmental Determinants of Diabetes in the Young (TEDDY) studies examined the gut and plasma virome of children with type 1 diabetes, respectively^13,14^. Additionally, a recent study examined the gut virome of 11 infants with type 1 diabetes^15^. However, data on vertebrate-infecting viruses from these studies remain difficult to interpret, partly due to the limited sensitivity of conventional virome sequencing caused by the overwhelming abundance of host and bacterial nucleic acid in most clinical specimens^16^.

Here, we used Virome-Capture-Sequencing for Vertebrate-infecting viruses (VirCapSeq-VERT) as an enhanced and unbiased method to characterise the virome of children with IA. With sensitivity on par or greater than quantitative real-time PCR (qPCR), VirCapSeq-VERT utilises ~2 million capture probes covering entire genomes of every known vertebrate-infecting virus for enrichment prior to high-throughput sequencing. This produces up to 10,000-fold increase in viral reads compared to conventional methods^16^.

## Methods

### Subjects and samples

This case-control study was nested within the Australian Viruses In the Genetically at Risk (VIGR) prospective birth cohort of children with ≥1 first-degree relative with type 1 diabetes^4^. We examined the virome of 45 cases with IA, defined as seropositivity for ≥1 islet autoantibodies (GAD65, IA-2 and IAA) above the cut-off (0.6 units/mL, 0.8 units/mL, 53 nU/mL, respectively) in two consecutive visits. Every case sample was paired with an age and gender matched persistently IA-negative control sample (Table 1). HLA class II information was available for 86/93 children, of whom 30 (17 cases, 13 controls) were categorised as having a type 1 diabetes risk genotype.

**Table 1:** Summary of study participant characteristics.

Characteristic	Case (n = 45)	Control (n = 48)	*P* value
Male, n (%)	25 (56)	27 (56)	0.95
Risk HLA Class II genotype†, n (%)^a^	17 (38)	13 (27)	0.27
DR3/DR4^b^	8 (18)	3 (6)	0.19
DR3/DR3^b^	0	1 (2)	0.50
DR4/DR4^b^	2 (4)	3 (6)	0.65
Mean age at time of sampling (SD)	5.7 (3.7)	5.4 (3.5)	0.64
**Relative with type 1 diabetes**, **n (%)**^**c**^			
Mother	17 (38)	25 (52)	
Father	14 (31)	15 (31)	
Sibling/s	15 (33)	9 (19)	
Progression to type 1 diabetes	3	0	
Samples, n	91	91	
Stool	32	32	
Plasma	59	59	

^*^Any of the following Human Leukocyte Antigen (HLA) genotypes: (1) HLA-DQB1*02/DQB1*0302; (2) HLA-DQB1*0302/x (x not DQB1*02, DQB1*0301 or DQB1*0602); (3). HLA-DQA1*05-DQB1*02/y (y not DQA1*0201-DQB1*02, DQB1*0301, DQB1*0602 or DQB1*0603); (4) HLA-DQA1*03-DQB1*02/y (y not DQA1*0201-DQB1*02, DQB1*0301, DQB1*0602 or DQB1*0603).

^a^HLA Class II information not available for 3 cases and 4 controls.

^b^Precise HLA-DRB1 and -DQB1 typing available for 48/93 participants (26 cases, 22 controls).

^c^One case and one control child had both a parent and sibling with type 1 diabetes.

Overall, 64 faeces and 118 plasma collected and stored at −80°C between 2006–2015 were analysed. Of these, 32 faecal samples from 20 cases collected at seroconversion and/or 15 ± 6 (mean ± s.d.) months prior, and 59 plasma samples from 41 cases collected at seroconversion and/or within 13 ± 4 (mean ± s.d.) months prior were examined; of available samples this concerned always the one(s) collected closest to seroconversion. Of 93 participants, 25 were included in both the gut and blood virome analysis (16 cases, 9 controls). There were 26 visits at which both sample types were collected (Supplementary Table 1). The project was approved by the Sydney Children’s Hospital Network Human Research Ethics Committee (HREC#12SCHN225) and all experimental methods were performed in accordance with the relevant guidelines and regulations. For all child participants in the study, informed consent was provided in writing by a parent or guardian on the child’s behalf, consenting for research participation, collection of biological specimens and use of clinical data in this publication.

### VirCapSeq-VERT

Performed as previously described^16^ with following modifications. Total NA was extracted from 235 μL of plasma using the MagMAX Isolation Kit on KingFisher (ThermoFisher). For faeces, 30% (w/v) suspension in 1 x PBS was centrifuged (5 min, 13,000 x g) and 175 μL of supernatant was used. Purified NA was amplified using the Whole Transcriptome Amplification Kit 2 (Sigma-Aldrich)^17^. Products were purified using the ChargeSwitch-Pro Kit and quantified by PicoGreen (ThermoFisher). Capture-compatible libraries were prepared with KAPA Hyperplus (Roche) and pooled (20 and 24 libraries/pool for faeces and plasma, respectively).

Reads were de-multiplexed, filtered (primer and host sequences) and *de novo* assembled. Contigs and unique singletons were homology searched at both the nucleotide and protein level^16^. Correcting for index cross-talk, 0.1% of the highest read count for each virus was subtracted across all samples within each pool. Two positivity thresholds applied: (1) 100 viral reads matched at the species level, distributed randomly across the target sequence; (2) 50 viral reads per 100,000 raw reads^13^. Former threshold was empirically established based on its proximity to the detection limit of qPCR in whole blood^16^ and faeces (Supplementary Fig. 1).

### Statistical analyses

Participant characteristics and virus frequencies compared using Chi-squared and Fisher’s exact tests, respectively. Differential abundance was examined by edgeR^18^, using a matrix of read counts encompassing all samples and detected viruses. Before conversion to counts per million, each matrix entry had 1 added to avoid issues with division by, or log function of 0. Data normalised using the Relative Log Expression method with respect to library size^19^. Common and tag-wise methods were used to estimate the biological coefficient of variation. Samples were divided into case and control groups, and the “exact” test was used to perform hypothesis testing^20^. *P* values were adjusted to control false discovery rates^21^.

### EV and Multiplex qPCR

First strand cDNA was synthesised using SuperScript III (Invitrogen) with random hexamers and 8 µL total NA. qPCR performed with EV-specific primers F: 5′-TCC TCC GGC CCC TGA ATG CG-3′ and R: 5′-ATT GTC ACC ATA AGC AGC CA-3′, and probe 5′-[6FAM]TGT GTC GTA ACG GGT AAC TCT G[BHQ1]-3′ on the LightCycler 480II (Roche), using 4 µL cDNA, 12.5 µL 2X Sensimix II Probe (Bioline), 0.25 µM probe and 0.5 µM of each primer. Cycle conditions: 95 °C 5 min, 40 cycles 95 °C 5 s; 55 °C 45 s; 68 °C 45 s. Norovirus, astrovirus, rotavirus and sapovirus were assayed with the Allplex™ GI-Virus Assay kit (GI9701Y; Seegene) on the CFX96™ (Bio-Rad).

### Infection history

Acute infections, illnesses or symptoms indicative of potential viral infection experienced by children between study visits were collected with the corresponding dates by parents and provided to study personnel at every visit (usually every 6 months), starting from age 6 months. The association between virus positivity determined by VirCapSeq-VERT and past infection was examined using Chi-squared analysis on infection history recorded within 3 months preceding sample collection.

## Results

### Virus positivity determined by VirCapSeq-VERT

**Figure 1:**
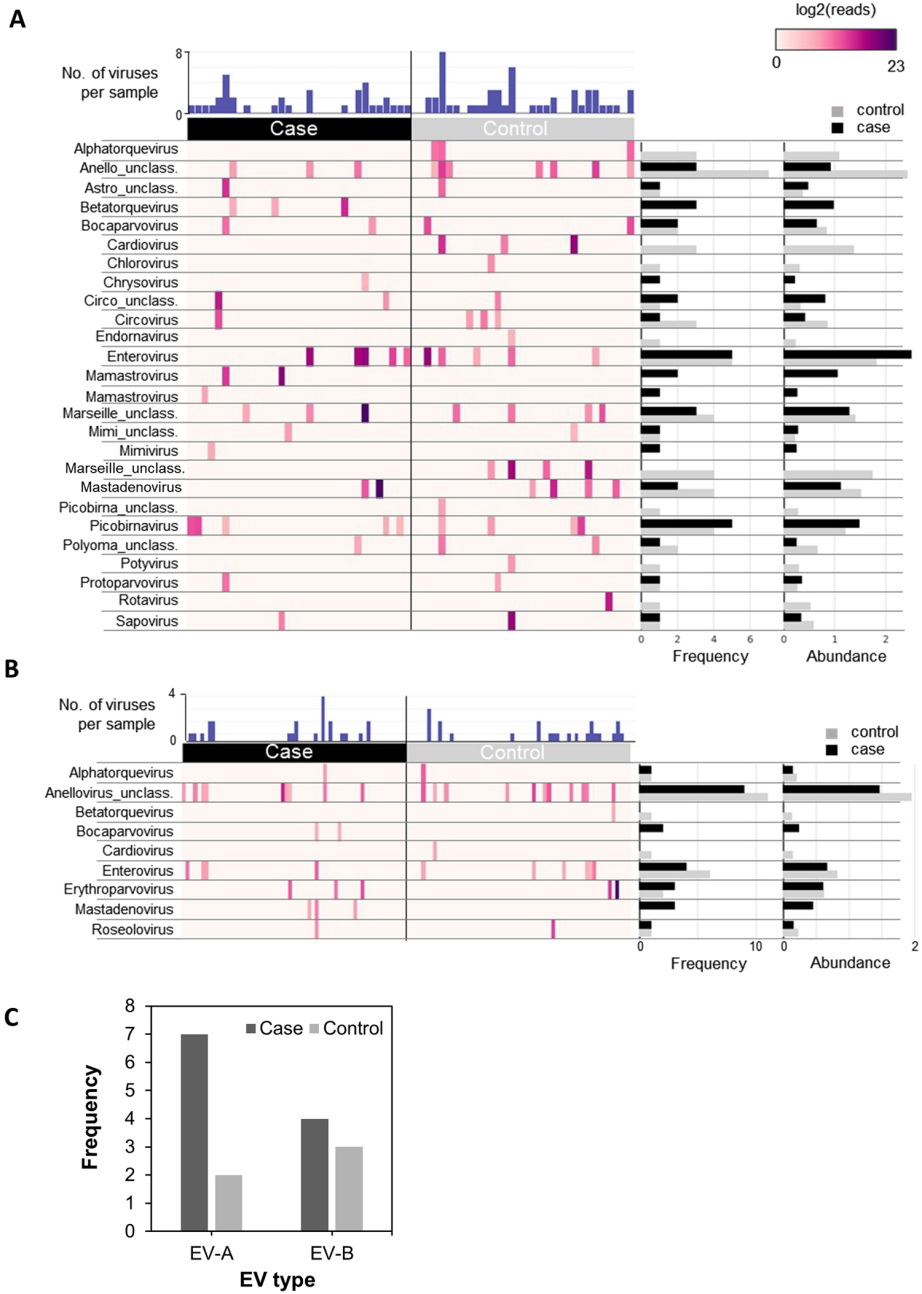
Viruses detected by VirCapSeq-VERT. (**A**) Heatmap of viral reads (log2 scale) detected in 32 case and control faeces (n = 64). Only viruses with ≥100 reads matched by BLAST at the species level were included and represented at the genus level. Number of viruses detected per specimen, frequency of each virus within the case/control group, and the mean log read counts (Abundance) are summarised by bar charts. (**B**) Heatmap of viral reads detected in 59 case and control plasma specimens (n = 118). (**C**) Frequency of EV-A and EV-B types sequenced from faeces of case and control children.

We characterised the virome of 93 children (45 cases with IA and 48 matched controls; Table 1) in the VIGR cohort [8]. Setting 100 reads as the positivity threshold, 75% of faeces (48/64) and 38% of plasma (45/118) were positive for ≥1 vertebrate-infecting virus (Fig. 1A,B), and 62% of children (58/93) were virus-positive. Of them, 43% reported a history of infection within 3 months prior to sample collection compared with 11% of virus-negative children (P = 0.002). There was no difference in virus positivity between cases and controls in the gut (66% vs 75%; P = 0.59) or plasma (41% vs 36%; P = 0.70), even when samples collected before and at seroconversion were analysed separately (Supplementary Table 2). We found no difference in virus positivity between children with type 1 diabetes risk HLA class II genotypes vs without (60% vs 68%, OR 0.71, 95% CI 0.30–1.78, P = 0.49).

In total, 28 viral genera were detected (Fig. 1). Anellovirus, EV and picobirnavirus were the most frequent viruses in faeces (Fig. 1A). In plasma, anellovirus, EV and erythroparvovirus were most frequent (Fig. 1B). Cardioviruses and noroviruses were detected exclusively in controls. There were minor differences in the frequency of specific viruses between cases and controls, but none reached statistical significance. In 19 children that had faeces and plasma collected on the same visit, EV was the only virus detected in both samples from the same timepoint (Supplementary Table 1); although only in 1/26 visits.

The prevalence of EV in faeces was identical between cases and controls. However, at the species level, EV-A and EV-B types were more frequent in cases (Fig. 1C). In some samples, multiple EV types were detected (Supplementary Table 3). In one case faecal specimen, reads matched to five different EVs (Rhinovirus C, ECHOvirus E30, Coxsackievirus B3; CVB3, CVA5 and CVA6) with 25–100% reference genome coverage and high sequence identity (>95%), which may indicate co-infection and/or recombinant genomes^22–24^. Presence of EV was also shown by qPCR in 5/5 EV-positive case faeces and 3/5 control samples (Supplementary Fig. 1). One negative qPCR result (KWK-291) was due to the presence of rhinovirus (Supplementary Table 3), not targeted by the qPCR. Norovirus, sapovirus and astrovirus positivity in faeces was likewise confirmed by multiplex qPCR (Supplementary Fig. 1).

**Figure 2:**
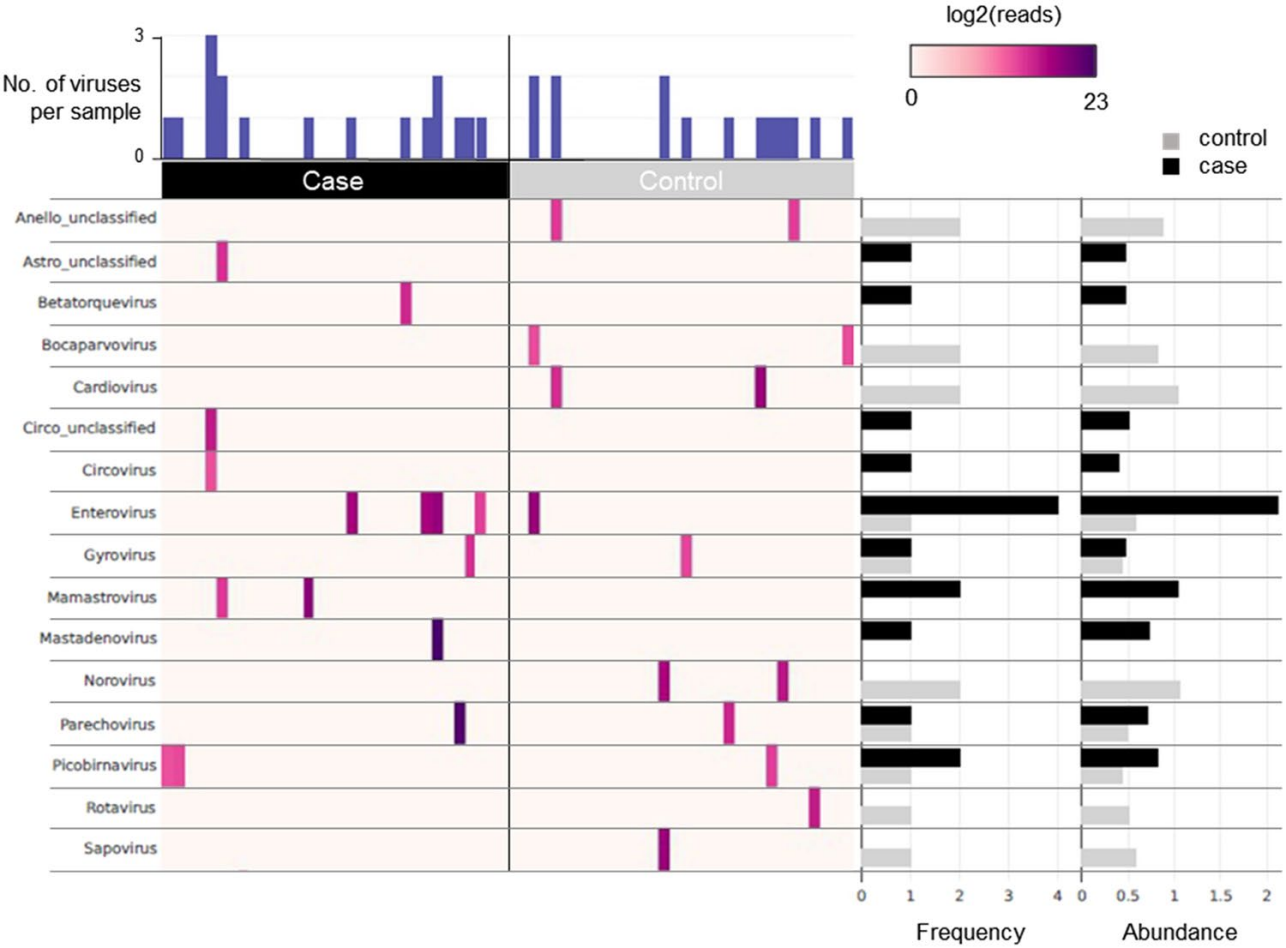
Viruses detected by VirCapSeq-VERT in faecal specimens based on the 50 reads per 100,000 raw counts (50P100K) threshold. Heatmap of virus reads (log2 scale) detected by VirCapSeq-VERT in 32 case and 32 control faeces (n = 64). Viruses represented at the genus level. Number of viruses detected per specimen, frequency of each virus within the case or control group, and the mean log read counts (Abundance) are summarised by bar charts.

Previously, 50 viral reads per 100,000 raw reads (50P100K) was used to indicate virus positivity in the DIPP virome study^13^. Using this threshold, faecal virus positivity reduced from 75% to 36% (23/64) (Fig. 2). Despite ~50% reduction, this still resulted in a >3-fold higher positivity than reported in the DIPP study (10%; 10/96), supporting the notion of an enhanced sensitivity of VirCapSeq-VERT. Frequencies of most viruses remained unchanged, except for a noticeable decrease in the number of EV-positive control faeces, rendering EV 4-fold higher in the gut of cases.

We found no association between EV in faeces and the first seropositivity for IAA (OR 0.66, 95% CI 0.13–3.58, P = 1.00) nor GADA (OR 1.41, 95% CI 0.33–6.27, P = 1.00).

### Differential abundance of gut viruses

**Figure 3:**
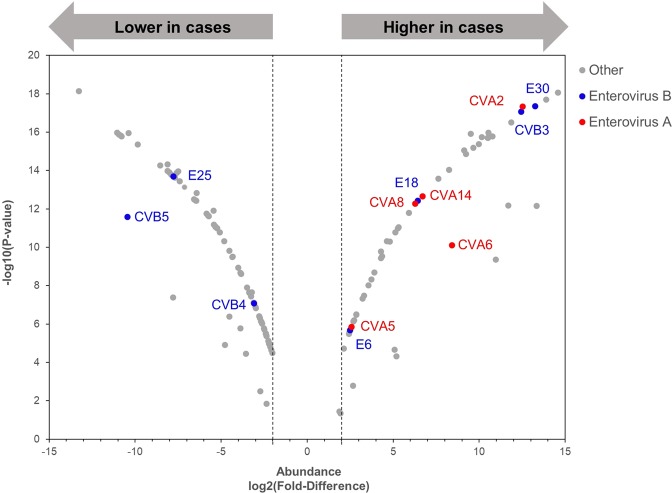
Higher abundance of enterovirus A in the gut of children with islet autoimmunity. Volcano plot of viruses differentially abundant between case and control in the gut. Only viruses with ≥2-fold difference (marked by dotted vertical line) and false discovery rate <5% (q < 0.05) as determined by edgeR are represented. Enterovirus A types represented in red: Coxsackievirus A2 (CVA2), CVA5, CVA6, CVA8 and CVA14. Enterovirus B types represented in blue: ECHOvirus E6 (E6), E18, E25, E30, CVB3, CVB4 and CVB5. All other viruses represented in grey.

In total, 129 viruses had ≥2-fold difference (*P* < 0.02) in abundance between the gut of cases and controls, with false discovery rate <5% (Fig. 3). One EV-A (CVA2) and two EV-Bs (ECHOvirus E30 and CVB3) were among the 10 most differentially abundant viruses (Supplementary Data), and every differentially abundant EV-A (CVA2, 5, 6, 8 and 14; Fig. 3 in red) was significantly more abundant in cases than in controls (P < 0.00001), even when samples collected before or at seroconversion were analysed separately (Supplementary Fig. 2).

## Discussion

This is the first examination all vertebrate-infecting viruses in a cohort at risk of type 1 diabetes using VirCapSeq-VERT. Analysis of faecal specimens from prior to or at seroconversion revealed significantly higher abundance of EV-A species in the gut of cases, strengthening the evidence that EVs contribute to the development of IA and that the risk for type 1 diabetes may be closely linked to viral load^25^. The lack of overlap between the gut and plasma virome is in keeping with the short duration of viremia following EV infection vs prolonged gut excretion^26^, corroborating the model that gut is a major regulator of early inflammation in type 1 diabetes^27^, and a reservoir from which EVs can spread into the pancreas^28,29^. Sufficient viral load may be important for EVs to establish persistent enteric infection, and thereby induce intestinal changes that contribute to the initiation of IA and the progression to type 1 diabetes. This is consistent with murine models showing the requirement of preceding intestinal inflammation for the development of β-cell autoimmunity^30^. Higher EV load may translate to higher replication and transmission to the pancreas, which can impose greater cellular stresses on pancreatic islets, contributing to inaccurate protein translation and production of aberrant insulin polypeptides that render β-cells immunogenic^31^.

The predominance of EV-A in VIGR children differed from previous associations of EV-B with type 1 diabetes^4,5^. However, it is consistent with the 10-year follow-up of 129 cases and 282 controls, which identified CVA2, A4 and A16 as the most common EV types in children with type 1 diabetes^32^. Furthermore, it fits the high prevalence of EV-A in natural circulation among infants^33^.

In this study, cardioviruses and noroviruses were detected exclusively in control samples. The biological significance of this is unclear, especially for cardioviruses which have unknown pathogenicity in humans^34^. Neither viruses have been associated with IA or type 1 diabetes. Indeed, RNA testing of human cardioviruses in 27 cases with IA vs 53 matched controls in the Norwegian longitudinal birth cohort study found no association between Cardiovirus in faeces and IA^35^.

Compared to case-control studies that used targeted detection methods on a selection of candidate viruses, our sample size is relatively small. However, it is comparable to other type 1 diabetes virome studies, and is the first to examine both the gut and plasma virome in children with IA. Low sample numbers potentially masked statistically significant differences in virus frequency between cases and controls. To ensure sufficient statistical power, future validation studies should examine a greater sample size and include multiple longitudinal timepoints preceding IA if possible. Nevertheless, we found a significant association between IA and higher gut EV-A abundance, and identified >100 additional viruses differentially abundant between the gut of cases and controls.

Another potential limitation is that participants were not matched for their HLA genotype. However, there is an increasing proportion of individuals with non-risk HLA genotypes developing type 1 diabetes, particularly in the Australian population^36–38^, and although a range of HLA genotypes are associated with increased susceptibility and persistence of some viruses, there is no evidence supporting their influence on the outcome of EV infections. Examination of over 7,000 longitudinal faecal specimens collected from 419 children with HLA-DR4/DR3 genotype and 373 without found no association between EV RNA detection and HLA risk^39^. Therefore, we consider it unlikely that our EV-A detection is primarily linked to HLA genotype.

In conclusion, studies into environmental triggers of type 1 diabetes have to date maintained a strong focus on identifying statistically significant differences in the frequencies of select viruses, especially CVBs^5^. Our findings highlight the importance of additionally examining the differential abundance of viruses. We demonstrate an association between IA and higher EV-A abundance in the gut of children, and shortlist additional viruses as potential contributors in the development of IA. These results should be validated in other cohorts with greater sample size and more longitudinal timepoints preceding IA, and likely candidates be targeted by mechanistic studies to determine if a causal link with type 1 diabetes exists, as it is paramount to define the full breadth diabetogenic viruses for future vaccine development.

## Supplementary information

Supplementary Info

Supplementary Data

## Data Availability

The datasets generated during and/or analysed during the current study are available from the corresponding author on reasonable request.
