# Supplementary material for: Higher abundance of enterovirus A species in the gut of children with islet autoimmunity
Source: Sci Rep. 2019 Feb 11;9:1749. doi: 10.1038/s41598-018-38368-8 (PMC6370883; doi:10.1038/s41598-018-38368-8)
Supplement: Supplementary file 1 — Supplementary Info [file 41598_2018_38368_MOESM1_ESM.pdf]

SUPPLEMENTARY INFORMATION

Higher abundance of enterovirus A species in the gut of children with islet autoimmunity

Ki Wook Kim[1,2¶], Jessica L. Horton[1,2¶], Chi Nam Ignatius Pang[3], Komal Jain[4], Preston Leung[5], Sonia R. Isaacs[1,2], Rowena A. Bull[5], Fabio Luciani[5], Marc R. Wilkins[3], Jacki Catteau[6], W. Ian Lipkin[4,7], William D. Rawlinson[1,2,8], Thomas Briese[4,9&] and Maria E. Craig[1,2,6,10&*]

[1] School of Women's and Children's Health, University of New South Wales Faculty of Medicine, Sydney, Australia

[2] Virology Research Laboratory, Prince of Wales Hospital Randwick, Sydney, Australia

[3] School of Biotechnology and Biomedical Sciences, University of New South Wales Faculty of Science, Sydney, Australia

[4] Center for Infection and Immunity, Mailman School of Public Health, Columbia University, New York, USA

[5] Systems Medicine, Inflammation and Infection Research Centre, School of Medical Sciences, University of New South Wales Faculty of Medicine, Sydney, Australia

[6] Institute of Endocrinology and Diabetes, Children's Hospital at Westmead, Sydney, Australia

[7] Department of Pathology and Neurology, College of Physicians & Surgeons, Columbia University, New York, USA

[8] Serology and Virology Division, South Eastern Area Laboratory Services Microbiology, Prince of Wales Hospital, Sydney, Australia

[9] Department of Epidemiology, Mailman School of Public Health, Columbia University, New York, USA

[10] Discipline of Child and Adolescent Health, University of Sydney, Sydney, Australia

[¶]KWK and JLH are Joint First Authors.

[&]MEC and TB are Joint Senior Authors.

*Corresponding author

Supplementary Table 1. Viruses detected in faeces and plasma collected from the same visit.

Visit	Case/Control	Viruses detected in Feces	Viruses detected in Plasma	Virus in common
1	Case	None	None	None
2	Case	EV	None	None
3	Case	EV	None	None
4	Case	Mimi_unclassified	None	None
5	Case	None	None	None
6	Case	None	None	None
7	Case	Mamastrovirus<br>Sapovirus	None	None
8	Case	Betatorquevirus	EV<br>Bocaparvovirus<br>Mastadenovirus<br>Roseolovirus	None
9	Case	Anello_unclassified<br>Betatorquevirus	None	None
10	Case	Astro_unclassified<br>Bocaparvovirus<br>Mamastrovirus<br>Picobirnavirus Protoparvovirus	None	None
11	Case	None	None	None
12	Case	None	None	None
13	Case	None	None	None
14	Case	Mastadenovirus	Anello_unclassified<br>Erythroparvovirus	None
15	Case	Picobirnavirus	None	None
16	Case	Picobirnavirus	Betatorquevirus	None
17	Case	Marseille_unclassified	None	None
18	Case	Betatorquevirus	None	None
19	Case	None	None	None
20	Control	Alphatorquevirus<br>Anello_unclassified<br>Bocaparvovirus	None	None
21	Control	Endornavirus<br>EV<br>Mastadenovirus<br>Norovirus<br>Potyvirus<br>Sapovirus	EV	EV
22	Control	None	None	None
23	Control	None	None	None
24	Control	Circovirus	Roseolovirus	None
25	Control	None	None	None
26	Control	Parechovirus	None	None

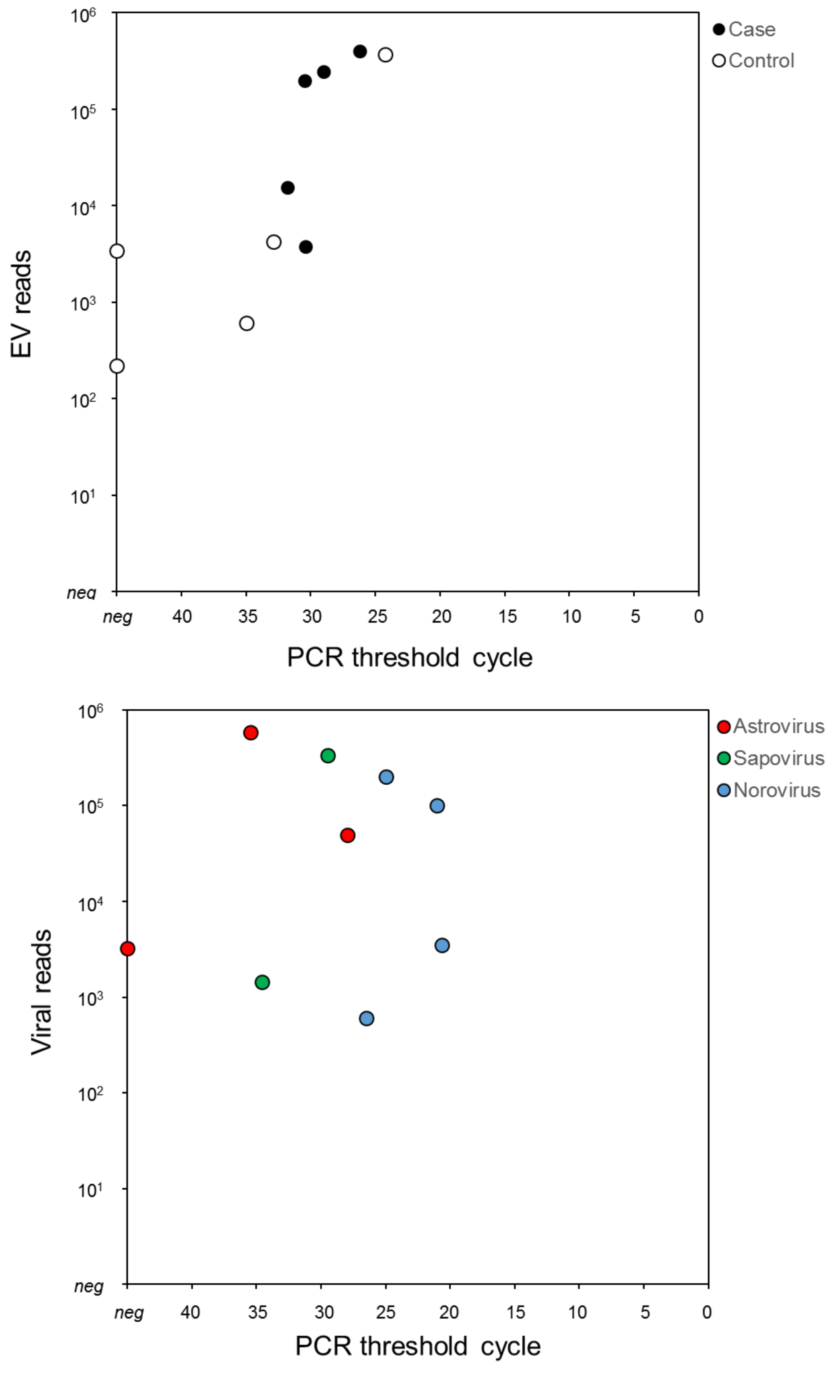

Supplementary Figure 1. Comparison of virus detection by VirCapSeq-VERT vs targeted real-time PCR (qPCR). (Top) Five case and five control faeces identified as EV-positive by VirCapSeq-VERT. Two of the control specimens tested negative (*neg*) by EV-specific qPCR. (Bottom) Multiplex qPCR of 10 faecal specimens identified as positive for astroviruses, sapoviruses and noroviruses by VirCapSeq-VERT. One astrovirus positive specimen tested negative (*neg*) by multiplex qPCR.

Supplementary Table 2. Virus positivity in faeces collected before and at seroconversion to IA.

Variable	n	Virus Positive (%)	Virus Negative (%)	*P* value
Case (before seroconversion)	14	9 (64.3)	5 (35.7)	0.70
Control	15	11 (73.3)	4 (26.7)	
Case (at seroconversion)	18	12 (66.7)	6 (33.3)	0.71
Control	17	13 (76.5)	4 (23.5)	

Supplementary Table 3. Reference genome coverage of EVs detected in faeces.

Sample	Enterovirus	Reference Genome (GenBank Accession)	Reference Sequence Length (nt)	No. Reads Mapped	Reference Coverage (%)
**Case**					
KWK-258	Coxsackievirus B3	KU574623.1	6,558	56,181	48
KWK-243	Coxsackievirus A2	KX156350.1	7,400	2,354	36
KWK-257	Rhinovirus C	JN815240.1	6,796	249	58
	ECHOvirus E30	EF066392.1	7,334	36,766	54
	Coxsackievirus B3	KR107057.1	7,399	5,293	39
	Coxsackievirus A6	KJ541158.1	7,412	84,400	100
	Coxsackievirus A5	AB114091.1	718	1,770	25
KWK-241	ECHOvirus E18	HM777023.1	7,413	2,555	49
KWK-267	Coxsackievirus A6	AB779616.1	7,434	40,292	25
	Coxsackievirus A2	KC879532.1	885	116,886	100
	Coxsackievirus A14	KP036483.1	7,400	3,720	14
	Coxsackievirus A8	KP765687.1	7,396	39,992	19
**Control**					
KWK-291	Rhinovirus A	JN798576.1	6,860	3,395	76
KWK-300	Coxsackievirus A6	KJ541158.1	7,412	208	12
KWK-424	Enterovirus A71	HQ647175.1	7,419	4,061	68
	Rhinovirus C	KF688606.1	6,928	136	13
KWK-426	Coxsackievirus B5	KT285015.1	6,937	203,415	42
	ECHOvirus E25	KX139460.1	7,436	2,103	13
KWK-259	Coxsackievirus B4	KX752784.1	7,372	258	20

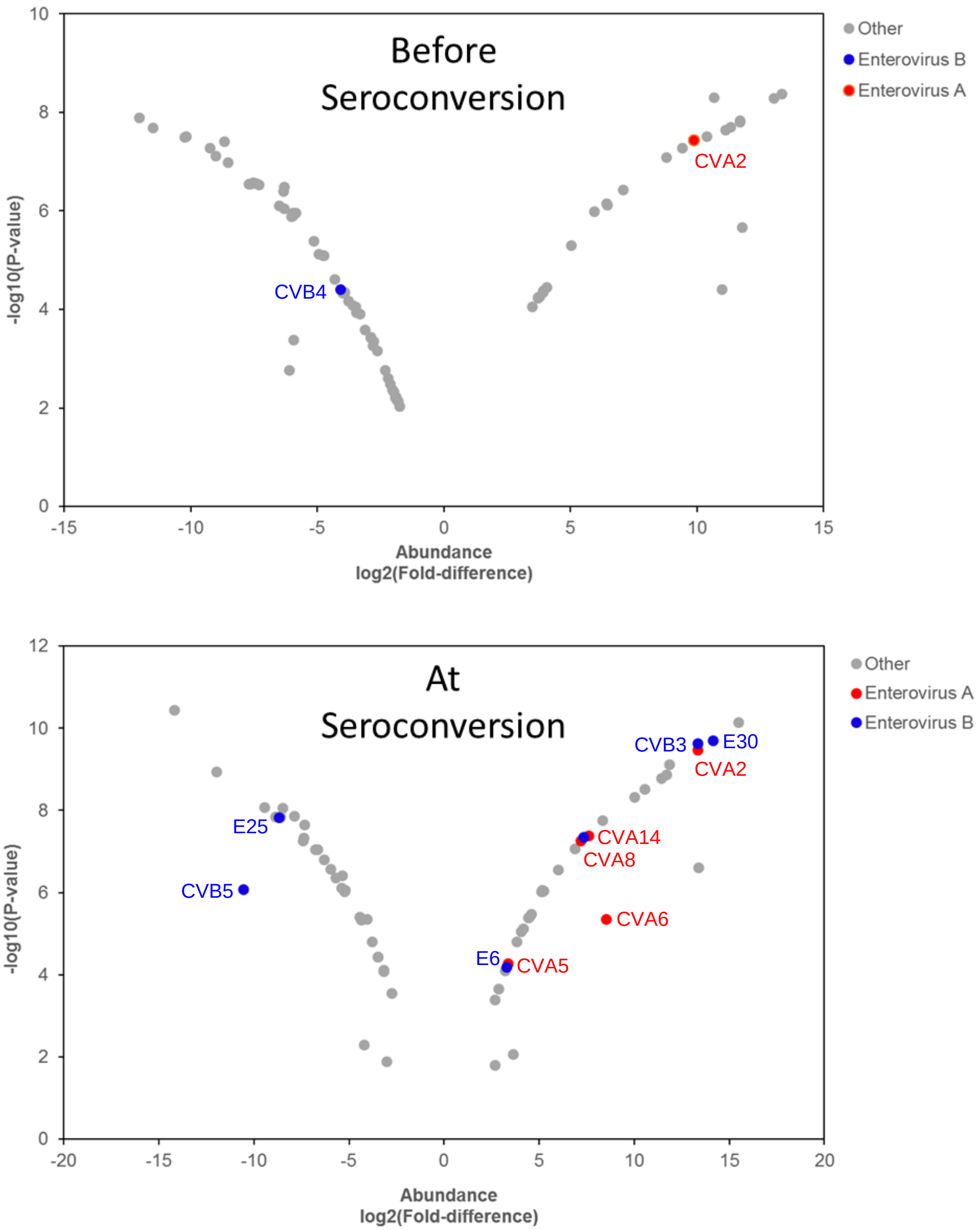

Supplementary Figure 2. Volcano plots of viruses differentially abundant between case and control children in faeces using the 100 read virus positivity threshold. Data from faeces collected before and at seroconversion to IA analysed and plotted separately. Only differentially abundant viruses with false discovery rate below 5% (q<0.05) as determined by edgeR are represented. Enterovirus A types represented in red, enterovirus B types in blue and all other viruses in grey.
